# Realistic Evaluation of the Integrated Electronic Diagnosis Approach (IeDA) for the Management of Childhood Illnesses at Primary Health Facilities in Burkina Faso

**DOI:** 10.34172/ijhpm.2022.6073

**Published:** 2023-01-01

**Authors:** Karl Blanchet, Vincent-Paul Sanon, Sophie Sarrassat, Arsène Satouro Somé

**Affiliations:** ^1^Geneva Centre of Humanitarian Studies, University of Geneva, Graduate Institute, Geneva, Switzerland.; ^2^London School of Hygiene and Tropical Medicine, London, UK.; ^3^Centre Muraz, Bobo Dioulasso, Burkina Faso.

**Keywords:** Digital Health, Mobile Health, Electronic Clinical Tool

## Abstract

**Background:** In 2014, Terre des Hommes (Tdh) together with the Ministry of Health (MoH) launched the Integrated electronic Diagnosis Approach (IeDA) intervention in two regions of Burkina Faso consisting of supplying every health centre with a digital algorithm. A realistic evaluation was conducted to understand the implementation process, the mechanisms by which the IeDA intervention lead to change.

**Methods:** Data collection took place between January 2016 and October 2017. Direct observation in health centres were conducted. In-depth interviews were conducted with 154 individuals including 92 healthcare workers (HCW) from health centres, 16 officers from district health authorities, 6 members of health centre management committees. In addition, 5 focus groups were organised with carers. The initial coding was based on a preliminary list of codes inspired by the middle-range theory (MRT).

**Results:** Our results showed that the adoption of the electronic protocol depended on a multiplicity of management practices including role distribution, team work, problem solving approach, task monitoring, training, supervision, support and recognition. Such changes lead to reorganising the health team and redistributing roles before and during consultation, and positive atmosphere that included recognition of each team member, organisational commitment and sense of belonging. Conditions for such management changes to be effective included open dialog at all levels of the system, a minimum of resources to cover the support services and supervision and regular discussions focusing on solving problems faced by health centre teams.

**Conclusion:** This project reinforces the point that in a successful diffusion of IeDA, it is necessary to combine the introduction of technology with support and management mechanisms. It also important to highlight that managers’ attitude plays a great place in the success of the intervention: open dialog and respect are crucial dimensions. This is aligned with the findings from other studies.

## Background

**Key Messages**

** Implications for policy makers**

- Successful diffusion of innovations requires combining the introduction of technology and support and management mechanisms.
- Health centre managers’ attitude plays a great place in the success of the intervention: open dialog and respect are crucial dimensions.
- The success of the diffusion depends mostly on qualified and trained healthcare workers (HCWs).

** Implications for the public**

The use of Electronic Computer-based Decision Support System (eCDSS) for the care of children under five, makes the global evaluation of these children during the consultation mandatory. In fact, using the electronic tool for the evaluation of a child lead to the following of all the steps necessary for the comprehensive care of that child. That improved substantially Healthcare workers’ (HCW’) adherence to Integrated Management of Chilhood Illness (IMCI) clinical assessment leading to some improvements in overall correct classifications but little or no improvement in overall correct prescriptions. Despite some limitations in the effect of the electronic tool on the correct prescription, we can conclude that the electronic device allows a better quality of care of these children.

Despite a large reduction in under-five child mortality (from 180 per 1000 live births in 1990 to 83 per 1000 live births in 2015), sub-Saharan Africa failed to reach Millennium Development Goal 4 target of 60 deaths per 1000 live births.^1^ In 1999, the World Health Organization (WHO) developed the Integrated Management of Chilhood Illness (IMCI) strategy.^2^ This strategy provides an algorithm to guide health workers through a systematic clinical assessment of sick children with the aim of improving the diagnostic classification and the treatment of these children^3-5^ and hence reducing mortality.^5,6^

 The IMCI guidelines follow a syndromic approach, whereby a limited number of selected symptoms and signs (with the highest sensitivity and specificity) are assessed. In fact, during an IMCI consultation, nurses assess first for general danger signs, they then assess the four main symptoms (cough or difficult breathing, diarrhoea, fever, ear pain) and the nutritional status. Assessing danger signs includes questioning the caregiver whether the child was unable to drink or breastfeed, was vomiting everything or had a history of convulsions. The child is then classified, according to the signs identified during the assessment of the child. The appropriate treatment covering the most likely diagnoses represented by each classification is identified. The IMCI guidelines incorporate counselling for caretakers and the identification of children who need to be referred to the next level of the health system.^7^

 However, effective implementation of IMCI is often constrained by poor adherence to the guidelines.^8-10^ Previous studies reported that adherence to the guidelines decreases over time due to inadequate initial training, shortage of staff and insufficient supervision.^11,12^

 Burkina Faso introduced the IMCI strategy in 2003. An evaluation conducted in 2013 found that only 24% of nurses working in primary care facilities had been trained in IMCI.^13^ Only 28% of children were assessed for three danger signs, and only 15% of children were correctly classified.^13^ On average only 6 out of 10 recommended tasks were performed. Forty percent of children judged to require referral by an IMCI expert were referred by healthcare workers (HCWs). While 91% of children with uncomplicated malaria received an artemisinin-based combination therapy, only 34% of children with pneumonia were correctly prescribed antibiotics and only 30% of children with diarrhoea were correctly prescribed oral rehydration salt.

 In 2014, Terre des Hommes (Tdh), a Swiss non-governmental organisation, together with the Ministry of Health (MoH), launched the Integrated electronic Diagnosis Approach (IeDA) intervention with the objective of improving adherence to IMCI guidelines in public primary health centres, in two regions of Burkina Faso, including Boucle du Mouhoun and Nord regions. The IMCI project was implemented at the primary healthcare level in health centres.

 The primary healthcare centres in Burkina Faso deliver a minimum package of services defined by the MoH of Burkina Faso comprising both preventive (eg, vaccinations, antenatal care, health education, and promotion of recommended nutrition, hygiene and safe water family behaviours) and curative measures (eg, treatment of common illnesses, minor surgery, supply of essential medicine, maternal and child consultations). The district health management team is in charge of supervising the Centres de Santé et de Promotion Social (CSPSs) and reporting routine data collected in them.^14^

 The IeDA intervention comprised five components: (1) An electronic computer-based decision support system (eCDSS) provided on tablets to primary health facilities and guiding HCWs through the IMCI national protocol during under-five consultations, from the clinical assessment of the child, through the classification, prescription, referral and counselling.

 The eCDSS tool guides HCWs in their clinical decisions and helps them respect the recommended IMCI protocol. The software by obliging the consultant to follow each step of IMCI protocol and fill each information box lead him/her to better adhere to IMCI guidelines. During the trial period, several versions of the software were deployed following feedback from users and stakeholders (patients, health staff, service providers and policy-makers – MoH, Tdh); (2) A six-day training course provided to HCWs on IMCI guidelines and the use of the eCDSS. During the last year of the trial, electronic learning modules with short videos were also available on tablets to support continuous training; (3) A quality assurance coaching system involving team meetings, two to four times a year, through which health district authorities and HCWs discussed solutions to their local issues (eg, organisation of care); (4) A supervision system including monthly visits by district management team to primary health facilities; (5) A health information system based on data collected through the eCDSS. During the last year of the trial, descriptive dashboards on under-five consultations were developed and shared with the health district authorities and HCWs.

 This intervention was the subject of the realistic evaluation.

 The IeDA intervention was evaluated using a mixed-methods study design composed of the following three interlinked studies^7^: (*i*) a stepped-wedge trial to evaluate the effect of IeDA on adherence to IMCI guidelines in primary health facilities (give the reference); (*ii*) a cost-effectiveness analysis to assess the value for money of the delivery of IeDA; and (*iii*) a realistic evaluation to understand the implementation process, the mechanisms by which the IeDA intervention leads to change and to identify factors that may affect these mechanisms at health centre and community levels.

 The stepped wedge trial^1^ showed that on average, 54% and 79% of clinical assessment tasks were observed to be completed by HCWs in the control and intervention districts respectively (cluster-level mean difference = 29.9%; *P* =.002). The proportion of children benefiting from correct classifications (ignoring the severity) was 73% and 79% in the control and intervention districts respectively (cluster-level mean difference = 10.1%; *P* =.004). The proportion of children who received correct prescriptions in accordance with HCWs’ classifications were similar across arms, 78% in the control arm and 77% in the intervention arm (cluster-level mean difference = -1.1%; *P* =.788). The IeDA intervention improved substantially HCWs’ adherence to IMCI’s clinical assessment tasks, leading to some overall increase in correct classifications but to little or no improvement in correct prescriptions. These mixed results from the stepped wedge trial did not provide any indication on the acceptance of such a tool at primary health level both by nurses and community members. As a result, a realistic evaluation was conducted to complement the trial.

 The realistic evaluation aimed to contribute to: describe the changes brought about by IeDA; describe the perceptions of IeDA by the actors; and analyze the factors that influence the implementation of the intervention.

 The theoretical framework of the study was based on both the principle of realistic evaluation and the diffusion of innovation theory. Application of realistic evaluation with large-scale change programs in healthcare systems is sparse, despite the importance of realistic evaluation and recent calls for its greater use in healthcare.^15^ As transformational change is both unpredictable and iterative, we adopted an interpretive case study design, drawing on the principles of realist evaluation.^16^ Rather than focusing on whether or not the project “worked” such approach aimed at determining how the project was shaped, enabled, and constrained by interaction between the context of the program and the chosen mechanisms of change.^15^

## Methods

###  Study Area

 This study was conducted in Burkina Faso, in West Africa. It measured the effect of the IeDA intervention on health systems in two regions of Burkina Faso, namely Boucle du Mouhoun and Nord regions. Both regions have 6 districts. Boucle du Mouhoun region has 226 CSPS for 1976 217 inhabitants including 18.58% children under five years.^17^ Ninety percent of children benefited from IMCI-based consulations.^17^ As far as the Nord region is concerned, there are 193 CSPS for 1 632 149 inhabitants, including 19.20% children under five years. Eighty-six percent benefited from IMCI-based consultations.^17^

 This study was conducted in eleven districts. Seven were intervention districts, including three in Boucle du Mouhoun region (Tougan, Solenzo, and Toma) and four in the Nord region (Yako, Séguénéga, Ouahigouya, and Titao). Four were control districts (Boromo, Dédougou, Gourcy, and Nouna). Tougan, Yako and Séguénéga consisted of the pilot districts where IeDA project has been implemented. In all districts, 10 CSPSs randomly selected for the stepped-wedge trial were used as study sites.

###  Theoretical Frameworks

####  Principles of Realistic Evaluation

 In the 1980s, Chen and Rossi developed the theory-based evaluation approach in answer to policy and program evaluation approaches that remained limited to before-and-after and input-output models or focused narrowly on methodological issues (method-oriented evaluation).^18^ This approach includes, among the most recent applications, the realist evaluation^16,18^ and the theory of change approach. Both approaches aim to open the black box between intervention and outcome. Realist evaluation is well-adapted to evaluating interventions in complex situations, such as change in social systems.^18,19^ While programs are the product of the foresight of policy-makers, their fate is always ultimately dependent on the imagination of practitioners and participants. Therefore, these visions are seldom fully aligned. Interventions never function endlessly, for all people, in the same way and under all conditions.^20^ Realistic evaluation is used to explain ‘what works, for whom and in what circumstances. For Pawson and Tilley,^16^ ‘what works’ is not of itself a helpful question as: “programs work (have successful ‘outcomes’) only insofar as they introduce the appropriate ideas and opportunities (‘mechanisms’) to groups in the appropriate social and cultural conditions (‘contexts’).”

 The task for the researcher is to distil the key potential mechanisms (M) and contexts (C) and examine how they interact in practice. That consist of theorising possible C+M combinations and explore the ways in which real-life experiences reflect and differ from these theories.

 Mechanisms are defined as a combination of inputs provided by the project and stakeholders’ reasoning and behvaiour change in response.^20^ It refers to the ways in which change is brought about by any process in the implementation process.^20^

 Context refers to the conditions under which programmes are introduced and implemented.^20^ Considering that some contexts will support the programme theory and others will not, realistic evaluation takes on the crucial task of sorting out the contexts by answering the questions “for whom” and “under what circumstances” a program will work.^20^

 Outcomes are both intended and unintended consequences of programmes. They stem from the activation of different mechanisms in different contexts.^20^ The evaluation contexts (primary healthcare centres in Burlina Faso) we are working in is one in which relatively little is known about ‘what works in what circumstances’ in relation to the use of the eCDSSs. Therefore, a core task for the research is to draw on existing data to theorise what seem to be likely ‘change mechanisms’ and to use the empirical study to explore the presence or otherwise of these C+M combinations, to examine the nature of their interaction and their consequences, both in terms of outcomes but also in terms of facilitating greater awareness of sustainability issues.

####  Formulation of Our Middle-Range Theory 

 The middle-range theory (MRT) is a theory that explains all the observed uniformities of social change and on the other hand the minor but necessary working hypotheses. It describes the manner in which the intervention leads to which effect in which conditions^18,21^ and how an intervention program input intends to reconfigure the existing component elements to produce a desired transformation.^22^

 In our study we formulated our MRT on the basis of an explorative study of the pilot districts where IeDA was first tested and can be stated as: “the importance of the characteristics of the innovation as a driver for use and the importance of the facility setting as a physical and organisational structure. We also found indications that the perception by communities was a determinant factor that drived health providers’ behaviour to adopt and use IeDA.” These three aspects (characteristics of the innovation introduced, characteristics of the health facility and perception by communities) guided our MRT.

 A second source of inspiration was the unpublished realistic synthesis of 36 peer-reviewed papers we conducted in 2015 on the factors influencing the use of eCDSS. It highlighted the interrelation between the properties of the innovation itself within a specific organisational environment. The contextual factors that influenced negatively the use of eCDSS were: financial incentives; competing programmes; lack of knowledge and use of information technology; high clinician turnover; link of eCDSS to an ordering system; and individual patient preferences for treatment.

####  The Innovation Attributes

 Health systems are viewed as complex systems.^23,24^ Complex systems are systems with a high number of elements or actors that interact with each other in ways that are not always predictable following the introduction of an innovation (eg, a new health intervention).^25,26^

 Diffusion of innovation theory can help explain how the continuation of activities is related to the attributes of activities as innovations. Beyond the description of an innovation as a newness, Rogers.^27^ Rogers showed that innovations are characterised by five attributes:

1. Relative advantage - individuals assess innovations by comparing the expected advantage of the new initiative with the benefits provided by the previous one that it replaced;
2. Compatibility – an innovation is perceived as compatible when the new idea or technology introduced by the innovation is consistent with the mandate of the adopters or the adopting system and does not require significant modifications from the adopters^28,29^;
3. Complexity – the perceived difficulty in understanding a new idea or using a new technology. A complex innovation can also be an intervention which involves a high number of actors^29,30^;
4. Testability – the notion that an innovation can be tested on a small scale^31^; and
5. Observability – the degree to which the results of the innovation are visible.^27,30^

The innovation attributes were used as a framework to structure the findings of the study incorporating elements of information related to implementation and organisational culture.

###  Data Collection

 During data collection that took place between January 2016 and October 2017, various sources of data, including literature review, direct observation, semi-structured qualitative interviews and focus group were used.

####  Direct Observation

 The direct observation was used for an immersion in the context^32^ and better understanding of the reality. We systematically conducted observations lasting from 30 minutes to two hours in the selected health facilities involved in the study. Unannounced visits were also made to certain urban CSPSs during the day and at night in order to diversify the cases encountered. In addition, these moments of observation were accompanied by informal interviews to better understand the observed facts. The direct observations helped in understanding the therapeutic relationship between patients and nurses and nurse assistants, the organization of the health centers and the district authorities for the implementation of IeDA.

####  Qualitative Interviews

 Qualitative interviews, including semi-structured qualitative interviews and focus group provided evidence in relation to the perceptions of IeDA, in-depth opinions and understandings of actors intervening in IeDA.

 The semi-structured qualitative interviews were conducted with 154 individuals including 92 HCWs from health centres, 16 officers from district health authorities, 6 members of health centre management committees, 9 child’s caretakers/mothers (See Table 1).

**Table 1:** Number and Profile of Individuals Interviewed During the Realistic Evaluation

**Profile**	**Number**
HCWs	92
Health district officers	16
Comite de Gestion	6
Child’s caretakers/mothers	9
Drug stock managers	3
Village representatives	2
Community health workers	6
Regional health authority	1
Health centre maintenance officer	2
Traditional chief	2
MoH officers	6
Tdh	3
**Total**	**154**

Abbreviations: HCWs, healthcare workers; Tdh, Terre des Hommes; MoH, Ministry of Health.

 In addition, 5 focus groups (on average 11 people per group) were organised with mothers and carers.

 The interview guides for the semi-structured interviews and focus group addressed the changes introduced by IeDA, the of IeDA, the benefit of IeDA, and suggestions.

 The sampling procedure was chosen according to the objectives of the study: generating theories and concepts rather than generalising findings to a wider population. Therefore, a purposive rather than a probabilistic sampling method was deliberately used by the investigator.^33,34^ Purposive sampling is used when researchers “seek out groups, settings and individuals where … the processes being studied are most likely to occur.”^35^ It helped to involve in a balanced way all categories of actors involved in the implementation of IeDA project and diversify the sources of information. Additionally, the “snowball principle”^36^ was applied. In this case, the previous interviewees and informants help in identifying the other informants. The focus group were performed with homogeneous groups of child’s caretakers/mothers selected among the patients with the support of the HCWs.

###  Data Analysis

 The semi-structured qualitative interviews and focus group were recorded, fully transcripted and coded for a manual content analysis. The initial coding of data collected through semi-structured qualitative interviews, focus group discussions and review of documents was based on a preliminary list of codes inspired by the MRT and on additional ideas that emerged from the fieldwork. In a second round of analysis, some themes and patterns emerged. In order to structure them as CMO (context + mechanism = outcome) combinations, we found it useful to borrow categories from theory-driven evaluation.^37^ We described the intervention in terms of content and application, and the intended and actual outcomes. We drew on our qualitative interviews, observations and document analysis to differentiate the vision (what the team wants), the discourse (what they say) and the actual practice (what they do). We described the organisational climate perceived by staff in terms of procedures, structures and incentives.^38^ In order to indicate how the intervention worked, we analysed both the context and the intervening mechanisms and attempted to identify the essential conditions.

## Results

 Results are reported in terms of implementation evaluation, policy context, and the innovation attributes.

###  The Actual Intervention

 Based on the analysis of qualitative interviews and project documents, we found that the list of intervention components that really constituted what the intervention was about went beyond the original vision. The project was very dynamic experiencing several stages of changes mainly guided by the feedbacks received from users. Tdh put in place regular dialog mechanisms with healthcare staff in order to ensure that the evolution of the tool and project take into account users’ feedback.

 Between May 2015 and December 2017, the intervention evolved. Several activities and tools were added during the last year primarily to improve knowledge and data use for management and clinical care: (*i*) development of dashboards at health centre level; (*ii*) supply of a second tablet to larger health centres; and (*iii*) online learning modules on IMCI including short videos available on the tablets. The “Registre Electronique de Consultations” (REC) itself evolved several times during the project period experiencing several software improvements on the tablet and the backend of the tool (data analysis) resulting in several consecutive versions of the REC.

###  The Policy Context

####  Free Healthcare Policy Initiative

 During the course of the project, a new policy emerged, which directly affected the utilisation of health services at health centre level. A free healthcare policy for children under 5 was nationally launched in April 2016.^39,40^ This decree was one step towards universal health coverage for which willingness of the Government had been officially formulated in September 2015 with a Law establishing a compulsory Universal Health Insurance.^41^ Following the implementation of this policy, the number of consultations in health centres significantly increased. It is likely to have had an impact on the increased workload of healthcare staff in health centres and the systematic use of the eCDSS. The introduction of the new policy was followed by the start of the malaria season in June 2016. According to nurses interviewed, the period of adaptation to the new workload lasted around seven months as soon as the malaria season ended and they had time to reorganise their services. In other words, the utilisation of the eCDSS was not deeply and durably impacted by the new policy.

####  Staff Turnover

 IeDA was implemented in all primary health facilities including those located in rural remote areas, where HCWs usually do not want to spend more than a few years and where staff turnover was anecdotally said to be high. For example, in Titao district, it was reported that up to 95% of newly transferred staff were HCWs coming straight from nursing school with no primary experience. During the semi-structured qualitative interviews, district managers estimated that newly arrived staff worked during an average of three years in the district before asking to be transferred to another district. Staff turnover was also seen as a challenge for Tdh who worried about training staff and sustaining the utilisation of REC in each health centre. In July 2017, out of all HCWs working in the four districts where IeDA had been implemented, 31% of them (62 out of 198) who were asked to use the eCDSS had not benefited from any IMCI/REC training. This was exclusively explained by staff turnover: nurses who had been trained by the IeDA project had been transferred to other districts and replaced by staff who had not received the initial IMCI/REC training. To triangulate the information, all 40 health centres were surveyed to understand staff turnover. It was found that 36% of nurses had been changed within the last 12 months, period of time corresponding to the first IMCI/REC training. District managers confirmed that the rate is constant every year. This suggests that every 12 months, around 40% of the nurses or midwives move to another facility (most of the time outside the district).

####  Innovation Attributes 

 The IeDA intervention and more specifically the technological innovation, the eCDSS, provided to nurses on tablets was analysed in relation to Rogers’ attributes: comparative advantage, compatibility, complexity, testability and observability.

###  Comparative Advantage

 In terms of comparative advantage, the REC was compared by HCWs to the previous situation where only paper-based version of the IMCI was available. We learned from the stepped wedge trial that IMCI paper-form was used for 68% (916/1343) of the consultations in the control arm, while the REC was used in nearly all consultations (97%, 674/694) in the intervention arm. The HCWs highlighted the advantages of the REC, which is described as a tool covering several functions. The REC was well accepted by HCWs and became a routine tool in their practice to the point that HCWs contributed to the maintenance of the tool, regular synchronisation and did not hesitate sometimes to use their own money to cover internet costs. The district officers as well as the HCWs recognised that the tool is well designed and enables the HCWs to directly have access to the protocol without searching for the right information. As a result, HCWs felt more confident in their own classification and prescription.

 “*If you directly register the child in the REC, it [the REC] provides the classification, the medicine you need to prescribe, even the dose. So no need anymore to search in the documents [ ie, IMCI paper protocol]. So to me, it is much easier like this: you ask questions, record the answer and this is finished. You get the treatment and the prescription. Huge advantage!*” (Semi-structured interview with HCW 1).

 “*Without the REC, there are many questions we used to forget. But here, all the questions are listed and you cannot skip any of them. So to me, I think that we better manage patients. For example, when a child comes with a simple malaria, you can without the REC forget to identify anaemia ” *(Semi-structured interview with HCW 1).

 The eCDSS was also seen as a dynamic tool, which evolved with the national policies through low cost uploads. During the course of the project in 2016, a revised version of the national IMCI protocol was introduced by MoH. The protocol was then supposed to be rolled out by the MoH, which required dissemination of the document and ideally refreshers for all health staff. With the eCDSS, a revision of the protocol in the software and the upload of the revised protocol on each tablet were the only tasks necessary to a full roll out of the revised protocol.

 From the perspective of the HCWs, nurses or nurse assistants, the eCDSS also represented a tool supporting continuous development through the eLearning tools. Indeed, in 2017 were introduced online training modules with short demonstration videos.

 “*For example, in terms of respiratory infections, to check whether a child has a stridor, you can click on the REC to watch a short video with a specific case of stridor. The REC provides a few more extra details on what information we need to check to confirm a stridor. They are plenty of details provided” *(Semi-structured interview with HCW 2).

 The REC is described by many community interviewees as a living entity with its own autonomy and decision power. As a result, the “machine” brings its own independent opinion on the top of the HCW’s opinion.

 “*It is the REC that helps quickly find the right products that are needed to treat my child when he is sick*” (Focus group with mothers).

 “*The machine gives more information than the nurse*” (Semi-structured interview with father 1).

 In a sense, the presence of the REC is reassuring for the community as it is a way to guarantee and triangulate the diagnosis provided by a nurse. To go further, it is as if the community had more trust in the REC viewed as generating a non-biased opinion:

 “*To me it is like a machine. It is a computer. This will diminish the errors. When I see some work done with a machine, I have no fear. I respect this work” *(Semi-structured interview with father 2).

 A second advantage of the REC is the capacity to generate a patient registry and even the medical history of the child. Thanks to the patient history function, the HCW can retrieve his/her consultation and ask further questions to the carer. Access to the medical history of the child is probably the most visible function from the perspective of the HCWs.

 “*When the child is here, you click here to see past treatments. You can see when he came and what reason. With the registry, it is very difficult. And we change registry all the time as soon as the pages are finished. But here, even one year later, you see everything” *(Semi-structured interview with HCW 2).

 Another important function of the REC is the centralisation and sharing of data. The patient registry is saved on the tablet, saved on a cloud and shared with district and national authorities.

 “*At the end of each week, data are sent to the district – very quickly – from the tablets without leaving the health centre. We can say that what we save is time” *(Semi-structured interview with townhall employee).

###  Compatibility

 In terms of compatibility, we investigated the compatibility with the infrastructure, the use of IMCI, the health team and the relationship patient/clinician.

 In terms of infrastructure, the REC did not create any specific challenge for the health centres, whatever the size of the health centres. The introduction of the REC systematically generated amongst the health team an inventory of equipment missing or not functioning and the list of essential medicines. For example, in many health centres, after the IMCI training and the introduction of REC, we observed the creation of oral rehydration therapy corners with plastic containers and oral rehydration solution.

 “*IMCI requires a consultation room dedicated to child consultations, which was possible in our health centre but we needed to move around furniture” *(Semi-structured interview with HCW 3).

 “*At the start, we through that the REC was asking for drugs that we do not have in stock. We then realised that these drugs were part of the essential list of medicines. We had to order them” *(Semi-structured interview with drug stock manager 1).

 In terms of team organisation, health staff realised that the use of REC was easier with several health agents involved than a single personnel. For example, one agent, usually the outreach health agent, stayed in the waiting room and take basic measures (weight and size). When possible, two agents managed the consultation as a team. One person close to the child and a second person guiding the consultation with the tablet through each step of the IMCI protocol and recording data on the tablet. We observed several times the involvement of one member of the health centre management committee when staff were overstretched.

 There were however situations when the use of REC was challenged by the population: when the agent was on his/her own and during the malaria season.

 “*If I take months such as September-October-November, when the waiting room is full of patients, people are vomiting, people are on the floor with fever, it is very challenging when staff is limited. The population would insult us if we are slow” (*Semi-structured interview with* HCW).*

 In terms of patient/clinician relationship, the REC introduced a new way of interacting with patients. One concern at the start of the project was that the REC would increase the physical distance between the patient and the HCW. In fact, we observed in several centres that one agent moved away from his/her desk to sit down next to the child in order to consult the child and ask questions to the carer. The HCWs noticed the satisfaction of the community in this new approach and felt a gain of trust from the community. When the REC was not functioning or out of battery, the community noticed it, asked for explanations and demanded the use of the REC during consultation time.

###  Complexity

 Complexity of REC was one of the main concerns the national policy-makers had. HCWs mentioned that the utilisation of REC becomes very complex when the system breakdowns. They also said that, it happened that in the middle of the consultation, the software froze or the system shut down deleting all information registered during the consultation. We also observed that in some health centres, nurses were using the paper registry as they had serious issues with the battery of the tablet. The point here is to highlight that the introduction had become so much part of routine practice that its absence due to a breakdown was noticed by the HCWs and disrupted the organisation of consultations.

 In addition to the system breakdowns, sometimes the use of the REC was not very compatible with the workflow leading some HCWs to use the paper-based IMCI forms instead. Other stopped using any IMCI document but filled patient information in the eCDSS only at the end of the whole consultation, reducing the REC to a repository function.

###  Testability

 The implementation of the IeDA project was progressive. Indeed, after the pilot districts, the project was extended to other health districts. Thus, the deployment of the project mechanisms was based on the achievements of the pilot phase while improving the implementation processes. This was marked by the use of several versions of the REC, on the one hand from the computer version to the tablet, on the other hand the addition of several functionalities such as a better presentation of the immunization calendar, the addition of a timer to count breathing, the addition of tools for reporting, the classification of malnutrition by weight/height ratio, the summary at the end of each module.

 In view of the advantages of the REC, most health workers, district managers and coordination structures would like to see the REC deployed in all health facilities. The start of a strategy to transfer the intervention’s achievements to the MoH was expected in December 2017. The transfer consisted of handing over the coordination of the activity to the State, through the MoH, which should take over the management of the intervention at the central, regional and district levels, resolve the technical problems encountered at all levels (CSPS, District, Regional, Central), and continue the development of the REC, including the addition of other functions maintain continuity in the conformity of the REC with the national IMCI protocol, ensure the reinforcement of the skills of the CSPS personnel through training, and finally, at the level of the statistical dimension, the State will take charge of the statistical tools that have been developed and will then ensure the availability of and access to medical data for all the components of the Ministry. The IeDA implementation process, from the pilot phase to the extension to other districts, supports the testability of the project mechanisms on a small scale (CSPS and pilot districts) before its scaling up (to the national level).

###  Observability

 In terms of observability (ie, the possibility for the users to perceive visible benefits), interviewees listed quite a few aspects. The degree to which the results of an innovation are visible to potential adopters were described by interviewee through observed positive impact/changes. In general, the participants to the study found that there is an improvement in the quality of care associated with the implementation of the project.

 First, the use of the REC positively affected the relationship between patients and caregivers, specifically, it created trust between users and health workers. This encourages them to attend more and more health facilities and to respect the appointments they are given.

 Secondly, to facilitate consultations, in most health centres, arrangements were made to bring the nurse and the patient closer together in the consultation room; thus, to avoid the consultation table being a barrier between the health worker and the users, the nurse sits next to the patient, which limits the back and forth between the table and the patient.

 Third, the intervention made it possible to provide the various health facilities with the medicotechnical equipment required for IMCI; in addition, as previously mentioned, for the intervention to be successful, there is renewed interest in monitoring the repositories in order to make the essential medicine available.

 Fourth, the HCWs realised that the use of REC lead to a more rational prescription of medicine and reduced over-prescription, which is usually the result from community pressure. The presence of the tablet provided vis-à-vis the community arguments a rationale for the HCW for not prescribing drugs when not necessary.

 Fifth, as part of the REC deployment, training on IMCI and CDR was provided, and the use of this tool allowed the caregiver to be continuously trained. Thus, the use of the REC strengthened the skills of health workers in the district.

 Sixth, the REC also strengthened collaboration and team spirit in the health facilities. In order to achieve the objectives, health workers changed the way they organized their care, and they regularly met to identify problems inherent in the use of the REC and find solutions.

 The use of the REC contributed to the improvement of the quality of consultations and thus to the improvement of the health of the population, as evidenced by the reduction of pathologies, the reduction of mortality rates and the large-scale respect of the implementation of the IMCI.

 Seventh, the quality of care approach promoted by MoH and Tdh went beyond the improvement of individual practice and behaviour change. A real support system was put in place engaging each level of the health system in the implementation and promotion of quality of care practices. This required the involvement of a wide range of actors ranging from national actors from all levels and sectors of the health system (different departments at MoH including family medicine, statistics and information) and international donors and United Nations agencies as well as non-governmental organisations and civil society organisations and individuals (opinion leaders, religious leaders). Many of these actors were involved and engaged at each stage of the project to share views on the next steps of the implementation and scaling-up of IeDA. The recognition of everybody’s voice created an atmosphere of mutual support and trust within health centres and between health centre staff and district health teams.

 “*The culture of performance and quality needs to start from the institutional level. We need to be able to support the institutional level, which means the national, district and health centre levels” *(Semi-structured interview with Tdh).

 The behaviour of health workers was also influenced by the new accountability system introduced de facto by the REC. Indeed, every HCW needs to log in every time he uses the REC. The information officer at the district level could easily retrieve this information in case of problem. This was a significant change in the Burkinabe public service culture as for the first time this information could be used to identify malpractice (if needed).

 The high level of commitment from a wide range of actors generated more legitimacy for the project and created a devolution of powers and responsibilities within the health system to monitor the quality of the services provided. Even most health facility managers had a sense that it was their responsibility to monitor the quality of the consultations performed by their team.

 “*The person who leads the consultation has to provide his personal details, which helps identify who is in charge of the consultation, so we know the proportion of consultations performed by nurses, as they are the ones who supposed to do it. And when there are problems, we can identify which person has difficult conduct correct consultations” *(Semi-structured interview with a district Officer).

###  Analysis of the Factors That Influenced the Implementation

####  CMO Configurations

 During the later phases of the analysis, we found that the adoption process can be grouped according to their key mechanism and this led to the description of parallel CMO configurations, each with their own outcome (Table 2).

**Table 2:** The three CMO Configurations Related to IeDA

**Context**	**Mechanism**	**Outcome **
C1. Availability of a support team to be responsive to healthcare staff questions.	M1. Promoting amongst HCWs “doing the right thing the right way” approaches.	O1. Notions of quality in childhood illnesses routinised during consultations.
C2. In health centres where the nurse is assisted by at least two other members (nurse assistants or outreach workers) and where management flexibility is allowed.	M2. Adaptability of roles before and during child consultations (including triage, weight and size measurements, consultation and counselling) to accommodate the adoption and use of the REC.	O2. Efficient organisation of the health team.
C3. Strong consensus amongst stakeholders on the benefits of introducing REC.	M3. Introducing at primary healthcare level the notion of individual accountability, belongness and responsibility and collective contribution to the wider system.	O3. Sustained use of REC as a routine practice with no interruption of the functioning of the tool.

Abbreviations: HCWs, healthcare workers; CMO, context + mechanism = outcome; REC, Registre Electronique de Consultations; IeDA, Integrated electronic Diagnosis Approach.

 The first CMO can be summarised as: *with support team responsive to healthcare staff questions and needs (C1), promotion amongst HCWs of “doing the right thing the right way” approaches (M1) in order to routinise notions of quality in childhood illnesses during consultations (O1)*.

 As described in the comparative advantage and observability sections, IeDA is trying to influence practice of HCWs by moving away from “simply doing.” In addition to the initial training, regular supervision was put in place to complement initial training with in service-supervision. This was accompanied by quality assurance sessions where staff in health centre were asked to find solutions as a team. There was also much attention for a clear role distribution within a health centre. The notion of teamwork was emphasized by the project management team recognising the value and role of each member, whatever the title and background. In summary, both “hard” and “soft” management mechanisms were used to influence the organisational culture. The former included task distribution between healthcare staff by task – pre- (eg, triage, child measurements), during (eg, consultation and prescription), and post- (eg, counselling) consultation tasks – and between clinical and administrative activities. The latter included initial IMCI/REC courses, peer pressure/support mechanisms and personnel development opportunities through eLearning modules. Availability of a support team to be responsive to healthcare staff questions and needs is an important context element and make possible the combination of all these management processes.

 The second CMO configuration can be summarised as follows: *a health centre team where the nurse is assisted by at least two other members (nurse assistants or outreach workers) and management flexibility is permitted (C2) can be better organised and efficient (O2) when the roles of each member can be adapted before and during child consultations to the adoption and use of the REC (M2).*

 As explained in the compatibility and complexity sections, consultations were organised considering the introduction of a new tool, the eCDSS, and quality assurance sessions. In order to be more efficient, a triage was conducted in the waiting room by a nurse assistant or an outreach agent who identified the children in critical condition and take child measurement (eg, size). This reinforced open relationships between health centre staff and contributed to solving practical problems and build solidarity between staff members. The quality assurance sessions were built around specific concrete issues experienced by the health centre team and elaborated realistic solutions and action points, which achievement depended on how the members will work as a team. In turn, it stimulates the feeling of perceived organisational system and team mechanism. The leadership and management style introduced by Tdh is perceived by health centre staff to be effective and supportive.

 The third CMO configuration can be formulated as *after creating strong consensus at all levels on the benefits of using REC (C3), sustaining the use of REC as routine practice (O3) requires introducing the notion of individual responsibility, belongness and accountability (M3). *

 A seen in the comparative advantage and observability sections, the members of the health centre, the primary users of the REC, had the feeling of belonging to a system that was wider than their health centre and contributing to a bigger enterprise than their own district. This was the result of early and ongoing engagement with a wide range of actors ranging from national and district authorities to opinion leaders at community level.^42^ REC users felt strong and wide consensus on the necessity of testing and using REC – a unique message sent by a multiplicity of key stakeholders influencing the environment of HCWs. The introduction of the notion of individual accountability in public services through personal login on the software also contributed to enhance a sense of individual responsibility and contribution to the wider system.

####  The New Middle-Range Theory 

 Our analysis identified three CMO configurations that indicate causal pathways between use of REC and sets of management practices, and we modified the MRT accordingly:

*The adoption of a computer-based decision support tool by health staff at primary healthcare will be enhanced by having a leadership focusing on building wide consensus from surrounding stakeholders (local and national authorities) on the benefits of using such an innovation and having a wide of actors fully and truly engaged in the directions the project could take. This necessitates a system promoting flows of information between all levels of the health system where transparency of information is valued.*

*The introduction of such innovation needs to occur in an environment flexible enough to provide space to staff make decisions on the distribution of tasks within the team in order to better adapt their work to the new situation. On the other hand, the innovation, REC, needs to be flexible enough to take into account the constant changing policy environment and the emerging needs and requests from its users.*

*The REC is adopted when perceived by users and district managers as being encompassed within a broader quality improvement strategy where health staff is sensitised to the importance of quality and their capacity to address quality issues at their own level.*

*The introduction of the REC needs to be accompanied by a supportive atmosphere and environment (including community and policy-makers support), which can be translated by peer support and district authorities support, and availability of support services responding to software or hardware issues. The supportive environment is based on reciprocity and acknowledges individual contributions to the wider system. Conditions for such environment to be promoted by a leadership that creates a decentralised decision space where initiatives are respected.*

## Discussion

 This study offers interesting insights on how the introduction of one computer-based decision support tool combined with management support practices created new work practices.

###  Lessons for Policy and Practice

 This project reinforces the point that in a successful diffusion of innovations (such as in the case of IeDA), it is necessary to combine the introduction of technology with support and management mechanisms. It also shows that in management of HCWs, it is important to mix different management practices. It is also important to highlight that managers’ attitude plays a great place in the success of the intervention: open dialog and respect are crucial dimensions. This is aligned with the findings from other studies. Mechanisms guidance change agents’ efforts. Change included finding and using evidence, involving service users in the modernization effort, developing the workforce, and expanding the range of services.^15^

 Our MRT highlights that the adoption of the REC is associated with a broader quality improvement strategy where health staff is aware of the importance of quality and their capacity to address quality issues by their own. This corroborates the findings from Marchal et al^18^ where the CMO configurations show that the availability of well-trained health workers and adequate funding seem to be critical contextual factors. The IeDA intervention included training, teamwork and supervision. This is consistent with Schein’s findings that teamwork and supervision serve as strong anchoring mechanisms for organizational culture.^18,43^

 Regarding the mechanisms, our findings relate to the analysis of Evans and Davis^44^ who situated the underlying mechanisms of high commitment management at the level of the internal social structure of the organisation. Such practices improve knowledge, practice and skills but also exert effects at the level of relationships between team members but also with line managers (in this case, the district health managers). Shared strategic vision contributes to a stronger organisational culture.^45,46^ Finally, a balanced management approach is costly, especially in management time (supervision, dialog, problem-solving sessions). It requires reasonable financial resources and a management capability to deal not only with administration but also with the less tangible issues of relationships, organisation culture and motivation of staff.

###  Methodological Lessons

 We used a realistic evaluation approach as we see health facilities as primarily being social entities. Pawson argued that realistic evaluation is well suited to investigate change in such social system.^16^ It provides an appropriate approach to increasing the external validity of case studies, the usefulness of case studies to managers and policy-makers by providing information that allows decision-makers to judge whether the lessons learnt could be applied elsewhere.^18,19^ However, appealing as it is, realistic evaluation poses a number of challenges for the researcher.

 The most critical issue is the attribution paradox. In complex systems, the behaviour of people is determined by many interlinked factors. Health professionals act under influence of their professional norms, social pressure, management interventions and their intrinsic motivation. Assessing the exact contribution of a set of management practices to overall performance is virtually impossible. What realistic evaluation can do is to stimulate the researcher to describe a detailed picture of the causal web that includes the multiple determinants and to categorise these as intervention, underlying mechanism and context. In our case, we argue that open dialog, training and support services are essential, but we don’t know which among these sets is the most important and in which setting. Rather than a logical-deductive exercise, generative causation of particular CMO alignments, is an interpretive task only achieved through negotiation and contestation.^15^ Consequently, evidence from realist evaluation can never be placed in the same categories as that produced by controlled experimental methods, due to their perspective on causality and the complexity of the topics on which they will be applied.^18^ The MRT is used in realistic evaluation to clarify key findings. A MRT cannot cover all possible explanations of change. A realistic evaluator does not pretend to provide the ultimate evidence that the intervention works. Rather, the MRT aims at enlightening the decision-maker, a process of utilisation of research that may be the most frequent in case of social research. In such cases, a pragmatic position should be taken whereby one tries to refine the MRT as much as practically possible with the explicit aim of providing options for improvement or scaling up rather than reaching a perfect understanding of the intervention as such.^47^

## Conclusion

 The realistic evaluation complemented the results of the stepped wedge trial and provided further insights on what worked and in which circumstances. Introducing a digital clinical tool in rural primary healthcare facilities in Burkina Faso has demonstrated to be a great initiative well appreciated by patients’ carers, authorities and healthcare staff. The introduction of the tool has requested investments in terms of supervision and coaching and harnessing team building and decision autonomy for decisions in each facility. This initiative really proves the interest of population and healthcare staff for tools consolidating clinical legitimacy. The use of realistic evaluation has showed being very valuable in documenting such real life issues and dynamics. Digital decision tools are now starting to being scaled up and such study design could serve as a foundation for the research community.

## Acknowledgment

 The research team would like to thank the staff at the MoH working in health centres who provided great support to the study, and the central support team of MoH and Tdh who provided support and technical input.

## Ethical issues

 The study was approved by the Ethics Committee of the Centre Muraz, Burkina Faso and the Ethics Committee of the London School of Hygiene and Tropical Medicine. All participants to the study provided written consent.

## Competing interests

 Authors declare that they have no competing interests.

## Authors’ contributions

 KB developed the methodology, analysed the data, drafted the paper, VPS and ASS developed the tools, collected data and analysed the data, SS analysed the data and all authors contributed to the writing up of the paper.

## Availability of data and materials

 All transcripts and notes are available at Centre Muraz, Burkina Faso.

## Funding

 The study was funded by Tdh, Switzerland.
